# Effect of Nb on *β* → *α*^″^ Martensitic Phase Transformation and Characterization of New Biomedical Ti-xNb-3Fe-9Zr Alloys

**DOI:** 10.1155/2021/8173425

**Published:** 2021-12-06

**Authors:** Syed Faraz Jawed, Chirag Dhirajlal Rabadia, Fahad Azim, Saad Jawaid Khan

**Affiliations:** ^1^Department of Biomedical Engineering, Ziauddin University, Pakistan; ^2^School of Engineering, Edith Cowan University, Joondalup, WA, Australia; ^3^Engineering Institute of Technology, Marquis St, Bentley WA, Australia; ^4^Department of Electrical Engineering, Ziauddin University, Pakistan

## Abstract

A new generation of Ti-xNb-3Fe-9Zr (*x* = 15, 20, 25, 30, 35 wt %) alloys have been designed using various theoretical approaches including DV-x*α* cluster, molybdenum equivalency, and electron to atom ratio. Afterward, designed alloys are fabricated using cold crucible levitation melting technique. The microstructure and mechanical performances of newly designed alloys are characterized in this work using scanning electron microscope and universal testing machine, respectively. Each alloy demonstrates monolithic *β* phase except Ti-35Nb-3Fe-9Zr alloy which display dual *α*^″^ + *β* phases. Typically, niobium acts as an isomorphous beta stabilizer. However, in this work, formation of martensitic *α*^″^ phases occurs at 35 wt % of niobium among the series of newly designed alloys. Furthermore, none of the alloys fail till the maximum load capacity of machine, i.e., 100 KN except Ti-35Nb-3Fe-9Zr alloy. Moreover, the Vickers hardness test is carried out on Ti-xNb-3Fe-9Zr alloys which demonstrate slip bands around the indentation for each alloy. Notably, the deformation bands and cracks around the indentations of each alloy have been observed using optical microscopy; Ti-35Nb-3Fe-9Zr demonstrates some cracks along with slip bands around its indentation. The Ti-25Nb-3Fe-9Zr alloy shows the highest yield strength of 1043 ± 20 MPa, large plasticity of 32 ± 0.5%, and adequate hardness of 152 ± 3.90 Hv among the investigated alloys. The Ti-25Nb-3Fe-9Zr alloy demonstrates good blend of strength and malleability. Therefore, Ti-25Nb-3Fe-9Zr can be used effectively for the biomedical applications.

## 1. Introduction

During the last two decades, titanium (Ti) alloys have been effectively employed in biomedical and high strength applications due to the combination of desired properties [1–3]. Many existing implant materials such as CP-Ti, Ti-6Al-4V, and Ti-Ni alloys demonstrate problems and limitations such as strength-ductility trade-off dilemma, stress-shielding effect, bio-incompatibility, and corrosion among others [4, 5]. Among different types of Ti alloys, *β*-type Ti alloys have been shown to be an effective solution for these aforementioned problems due to their excellent properties such as high strength, low modulus, and good biocompatibility among other qualities [6, 7]. The *β*-stability of newly designed alloys can be enhanced by alloying Ti with *β*-stabilizing elements such as Nb, Mo, Cr, and Fe [8]. Nb is a strong *β*-isomorphous element that reduces elastic modulus and increases *β*-phase stability, strength, shape memory effect, and superelasticity of titanium alloy [1, 9, 10]. Fe is a low-cost and a plentiful *β*-eutectoid element that has been employed as one of the main constituent in high-strength Ti alloys [11]. It was reported that 3 wt% Fe alloyed with Ti-25 wt% Nb displays a lowest modulus with differing concentrations (i.e., 1, 3, 5, and 7 wt%) of Fe elements [12]. Generally, Zr is regarded as a neutral element, but some studies have shown that the addition of Zr reduces the formation of *ω* phase in Ti alloys [13]. According to some studies, 8 wt% Zr is sufficient for complete retardation of *ω* phase in Ti alloys [14]. It was well documented that *β* phase stability and mechanical behavior of *β*-type Ti alloys are influenced by the presence of some martensitic phases such as *α*′, *α*^″^, and *ω* phases that are formed during quenching at room temperature [15]. Therefore, it is necessary to investigate the effects of the content of *β*-stabilizing elements on *β*-phase stability and characterizations of *β*-type Ti alloys. Accordingly, this work examines the effect of Nb on the formation of *β*⟶*α*^″^ martensitic phases. Moreover, the influence of Nb on microstructural and mechanical characterizations of newly designed Ti-xNb-3Fe-9Zr (*x* = 15, 20, 25, 30, and 35 wt %) alloys is also investigated. Notably, balanced combinations of alloying elements such as *β*-isomorphous (Nb), *β*-eutectoid (Fe), and neutral (Zr) elements that are alloyed with Ti are examined in this work. These elements are chosen in order to attain an effective blend of properties in newly designed Ti alloys for biomedical and high-strength applications.

## 2. Experimental Methods

**Figure 1:**
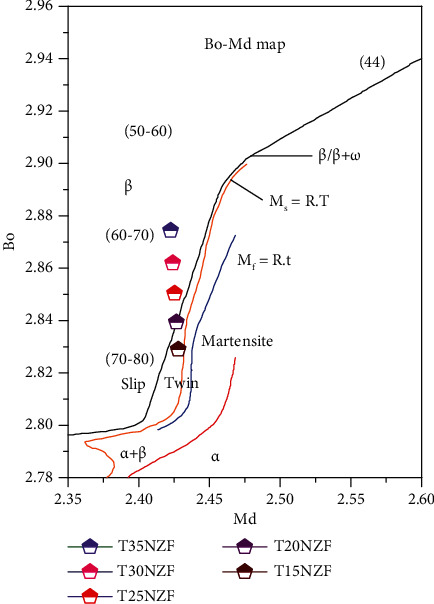
The Bo-Md phase stability diagram of the Ti-xNb-3Fe-9Zr alloys.

A novel series of Ti-xNb-3Fe-9Zr (where *x* = 15, 20, 25, 30, and 35 wt %) alloys were designed in this work and were hereafter named as TixFZ (where *x* = 15, 20, 25, 30, and 35 wt %). These newly designed TixFZ alloys were theoretically designed based on the DV-x*α* cluster technique developed by Morinaga et al. [16]. The phase stability map of newly designed alloys is presented in Figure 1. Note that the experimental results do not match with theoretical prediction results developed by Morinaga et al. However, some discrepancies have also been reported in the literature between the actual deformation mechanism and spotted location of each alloy on Bo-Md map [17]. Molybdenum equivalency (Mo_eq_) and electron to atom ratio (*e*/*a*) were also used for the predication of *β* phase stability of newly designed alloys [18, 19]. Further, in order to understand a deformation mechanism in the TixFZ alloys, the martensitic start temperature (Ms) was estimated for the TixFZ alloys using the following equation [20]:

(1) $$\begin{array}{c} M_{s}=1156–150\text{F}{\text{e}}_{\text{wt}\%}–96\text{C}{\text{r}}_{\text{wt}\%}–37{\text{V}}_{\text{wt}\%}–17\text{N}{\text{b}}_{\text{wt}\%}–7\text{Z}{\text{r}}_{\text{wt}\%}+15\text{A}{\text{l}}_{\text{wt}\%}. \end{array}$$

The values of theoretical parameters for all the TixFZ alloys are presented in Table 1. The theoretical design was kept in a manner based on these electronic parameters in order to get high *β* phase stability.

Following this step, the designed alloys were cast using the cold crucible levitation melting (CCLM) method. After this, the molten elements were solidified in the form of metal ingots that were flipped and remelted four to five times to ensure the homogeneity of the alloy mixture. Subsequently, circular rods of 3.5 mm diameter were extracted from the ingots for microstructural and mechanical characterizations. The circular rods were then cut into different lengths of flakes and then these flakes were ground and polished using silicon carbide grinding papers of up to 2000 grits and OP-S liquid on a polishing cloth, respectively. Afterward, surfaces of circular flakes were etched using Kroll's etchant containing 2 vol% HF, 6 vol% HNO_3_, and 92 vol% H_2_O for microstructural characterizations. Subsequently, microstructural characterizations were carried out on etched surfaces of circular flakes using an FEI verios XHR 460 scanning electron microscope (SEM) for all the TixFZ alloys. Accordingly, the grain size of various grains has been measured using ImageJ analysis, and average of at least ten measurements has been considered as the grain size of each alloy. Furthermore, the chemical analyses of produced alloys have been performed using energy-dispersive X-ray spectroscopy (EDS). The quantities of alloying elements (in wt %) and oxygen content (in ppm) are presented in Table 2. The results of chemical analysis are almost identical to nominal composition with low oxygen content (less than 2 wt %) presented in each TixFZ alloy. Phase characterizations were carried out using a PANalytical EMPYREAN diffractometer with Cu-k*α* radiation (*λ* = 0.15406 nm). The X-ray diffraction (XRD) spectra of each TixFZ alloy were obtained in the 2*θ* range from 20° to 90° with a scan step size of 0.02°. Further, the lattice parameter of body-centred cubic (bcc) *β* (*ɑ*_*β*_) TixFZ alloys was estimated using Nelson-Riley's extrapolation relation, i.e., ((cos2*θ*/sin*θ*) + (cos2*θ*/*θ*)).

For mechanical characterization, the ratio of length to diameter for all the TixFZ circular rods was kept as per requirements of ASTM standards, i.e., 1.5-2. Multiple independent uniaxial compression tests were performed for each TixFZ alloy using an Instron 5982 universal testing machine with a crosshead speed of 0.003 mm/s. Further, in order to achieve precised results, at least three samples of each alloy have been tested and average of their values has been considered for mechanical characterizations. Moreover, the new generation of alloys is subjected for compressive mechanical test because of the reason that bone and hard tissues are subjected to compressive loads rather than tension during daily living activities (DLA's) [7]. The values of true stress and true strain for compression testing were obtained using the following equations, respectively [21]:

(2) $$\begin{array}{c} \varepsilon^{\prime }=\ln\cdot \left(1–\varepsilon \right),\\ \sigma^{\prime }=\sigma \cdot \left(1–\varepsilon \right), \end{array}$$

where *ε*′, *ε*, *σ*′ and *σ* are compressive true strain, engineering strain, true stress, and engineering stress, respectively.

Vickers microhardness (Hv5) for all the TixFZ alloys was measured on the polished surface of samples using a Zwick-Roell hardness testing machine. An average of at least twelve indentations was taken at varied positions over the wide surface area of samples for all the TixFZ alloys. In order to analyze the elastoplastic deformation mechanism, deformation bands that formed around microhardness indentations were measured using a ZEISS Axiocam 208 color microscope.

## 3. Results and Discussion

**Figure 2:**
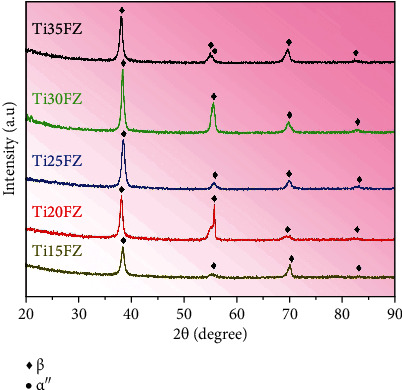
The XRD spectra of the Ti-xNb-3Fe-9Zr alloys produced via CCLM.

Phase characterizations for TixFZ alloys were executed using XRD, and their results are presented in Figure 2. It can be observed from Figure 2 that all the TixFZ alloys demonstrate a single bcc *β* phase, except for Ti-35Nb-3Fe-9Zr which displays dual phases, i.e., bcc *β* and orthorhombic *α*^″^ phases. The peaks of bcc *β* phase shifted towards lower 2*θ* angles when the amount of Nb in the TixFZ alloys is increased. This phenomenon can be ascribed to the higher Pauling's metallic radius of Nb (i.e., 0.1342 nm) then Ti (i.e., 0.1324 nm) [21, 22]. The lattice parameters of bcc *β* phase (*ɑ*_*β*_) are ranged from 0.3306 nm to 0.3337 nm for Ti15FZ to Ti35FZ alloys, respectively. The *ɑ*_*β*_ of the TixFZ alloys increases with alloying amounts of Nb, where this phenomenon can be attributed to Nb's high atomic radius [8].

**Figure 3:**
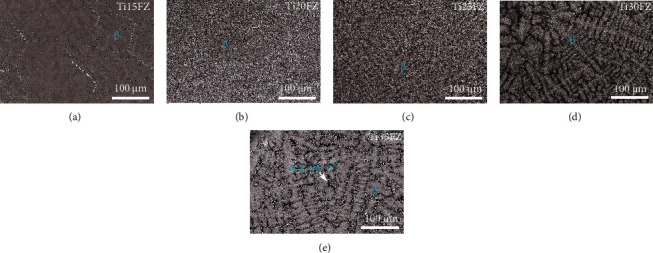
The backscattered SEM microstructural images for the Ti-xNb-3Fe-9Zr alloys.

The microstructural features displayed in Figure 3 demonstrate that all the TixFZ alloys exhibit monolithic *β* phase except for the Ti35FZ alloy. Generally, Nb is a *β* stabilizer element that reduces transition temperature and increases *β* stability [8]. However, it is interesting to note that Nb only stabilizes the bcc *β* phase from 15–30 wt% Nb in the TixFZ alloys. In contrast, an acicular orthorhombic *α*^″^ phase forms in the *β* matrix of Ti35FZ alloy. Hence, an excessive amount of Nb, i.e., 35 wt% results in a reverse martensitic *β*⟶*α*^″^ transformation in TixFZ alloys. Further, when an element possessing a high melting point (i.e., Nb) is added to Ti alloys, a dendritic substructure forms during solidification [9]. Accordingly, Figures 3(a)–3(e) clearly display that the density of dendritic substructure is raised with increasing amounts of Nb in the TixFZ alloys. The instability in *β*↔*α*^″^ transformation for a Nb enrich alloy is due to the occurrences of demixing process in alloy composition [23].

**Figure 4:**
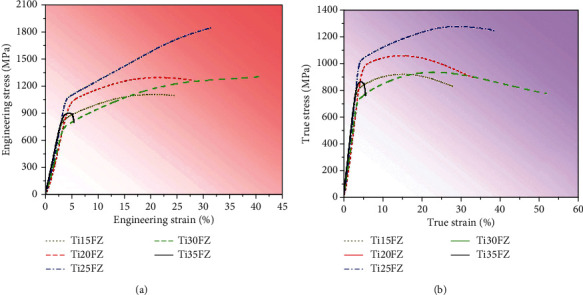
The compressive stress-strain curves for Ti-xNb-3Fe-9Zr alloys: (a) engineering stress-strain and (b) true stress-strain.

Mechanical properties are interrelated with their phase and microstructural characteristics [24], where Figures 4(a) and 4(b) show that almost all the TixFZ alloys, except Ti35FZ, did not fail during their compressive test because of the existence of only bcc *β* phase [14]. Contrastingly, Ti35FZ fails due to the presence of a dual-phase, i.e., martensitic orthorhombic *α*^″^ and bcc *β* phase [14]. It was reported that *M*_*s*_ less than 90°C indicates the superelastic nature of the alloy [25]. Hence, the reversible *β*⟶*α*^″^ transformation in Ti35FZ alloys demonstrates its superelastic behavior [26]. The Ti30FZ alloy demonstrates the highest total elongation of 38 ± 5% among all the TixFZ alloys. Notably, plasticity increases with increasing the Nb amount in single *β* phase containing TixFZ alloys, which occurs due to an increase in *β*-phase stability [27]. Cai et al. reported the formation of *α*^″^⟶*β* and unexpected counter-intuitive formation *β*⟶*α*^″^ during straining because of external stress [28].

**Figure 5:**
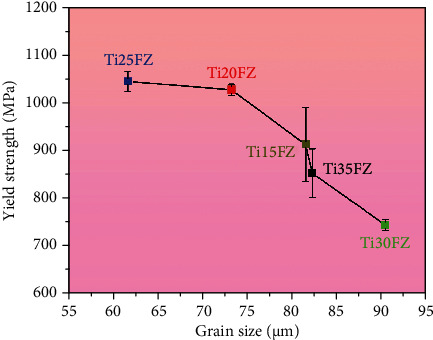
The yield strength (*σ*_0.2_) and grain size of *β* phase (*D*_*β*_) of Ti-xNb-3Fe-9Zr alloys.

Figure 5 demonstrates the relationship between yield strength (*σ*_0.2_) and mean *β* grain size (*D*_*β*_) for all tested TixFZ alloys. According to Hall-Petch relationship, the *σ*_0.2_ decreases with an increase in the *D*_*β*_ of a material, the Hall-Petch relationship satisfies for all the TixFZ alloys, i.e., the *σ*_0.2_ increases as the *D*_*β*_ decreases [29, 30]. The Ti25FZ alloy demonstrates the highest *σ*_0.2_, i.e., 1043 ± 20 MPa and the lowest *D*_*β*_, i.e., 62 ± 20 *μ*m among all the investigated TixFZ alloys.

**Figure 6:**
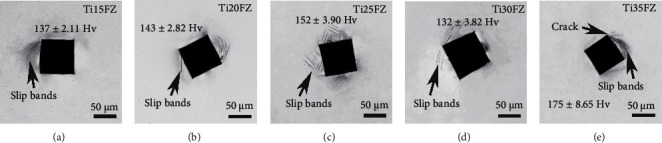
The optical micrograph of Vickers microhardness indentations and values of microhardness for all the Ti-xNb-3Fe-9Zr alloys.

Figure 6 displays deformation bands around the Vickers indentations along with values of Hv5 microhardness. It is evident from Figure 6 that the TixFZ alloys form slip bands around indentations. Moreover, crack originates from the corner of the Ti35FZ alloy indentation due to the existence of orthorhombic *α*^″^ phase [31]. Among all the investigated alloys, the Ti25FZ exhibits the greatest number of slip bands because of its highest yield strength [17, 32]. It was reported that hardness is directly proportional to the yield strength, where a similar trend has been found for all monolithic bcc *β* phase the TixFZ alloys [33]. The hardness of the Ti35FZ alloy is impacted by due to the presence of the orthorhombic *α*^″^ phase, where it demonstrates the highest microhardness value of 175 ± 8.65 Hv among all the investigated TixFZ alloys. The results of mechanical characterizations are in line with the results of phase and microstructural characterizations.

## 4. Conclusion

In conclusion, a new series of metastable *β*-type Ti-Nb-Fe-Zr alloys have been developed that demonstrate a combination of high strength and large plasticity. Almost all the investigated alloys demonstrated a monolithic *β* phase, except for the Ti-35Nb-3Fe-9Zr alloy. Interestingly, higher content of Nb (i.e., 35%) leads to a reversible martensitic *β*⟶*α*^″^ transformation in Ti-xNb-3Fe-9Zr alloys demonstrating its superelastic behavior based on their calculated values of Ms. Further, the Ti-25Nb-3Fe-9Zr alloy shows the highest yield strength of 1043 ± 20 MPa, large plasticity of 32 ± 0.5%, and adequate hardness of 152 ± 3.90 Hv among the investigated alloys. Among all the investigated alloys, the Ti-25Nb-3Fe-9Zr possesses the highest yield strength which indicates that it possesses the highest number of slip bands. Further, almost all Ti-Nb-Fe-Zr alloys except Ti-35Nb-3Fe-9Zr demonstrate significant plasticity due to the presence of monolithic *β* phase in their phase and microstructure analyses. By contrast, Ti-35Nb-3Fe-9Zr possesses dual-phase (i.e., *α*^″^ + *β*) which leads to its highest microhardness 175 ± 8.65 Hv among the investigated alloys.

## Figures and Tables

**Table 1:** The electronic parameters including average bond order ($\bar{Bo}$), average metal-*d* orbital energy level ($\bar{Md}$), molybdenum equivalency (Mo_eq_), electron to atom ratio (*e*/*a*), and martensite start temperature (*M*_*s*_).

Alloys	Compositions (wt%)	$\bar{Bo}$	$\bar{Md}$	Mo_eq_	*e*/*a*	*M*_*s*_, *K*
Ti15FZ	Ti-15Nb-3Fe-9Zr	2.8289	2.4225	11.666	4.20	388
Ti20FZ	Ti-20Nb-3Fe-9Zr	2.8393	2.4240	13.055	4.24	303
Ti25FZ	Ti-25Nb-3Fe-9Zr	2.8503	2.4253	14.444	4.27	218
Ti30FZ	Ti-30Nb-3Fe-9Zr	2.8620	2.4240	15.833	4.31	133
Ti35FZ	Ti-35Nb-3Fe-9Zr	2.8744	2.4225	17.222	4.36	48

**Table 2:** The values of chemical composition of alloying elements (wt%) and oxygen concentration (ppm) of TixFZ alloys.

Alloys	Chemical composition (wt %)	Ti (wt %)	Nb (wt %)	Fe (wt %)	Zr (wt %)	O (ppm)
Ti15FZ	Ti-15Nb-3Fe-9Zr	Bal.	14.95 ± 2.8	3.45 ± 0.7	8.80 ± 0.4	904 ± 2.56
Ti20FZ	Ti-20Nb-3Fe-9Zr	Bal.	19.78 ± 2.9	2.98 ± 0.2	8.62 ± 2.0	1065 ± 22.3
Ti25FZ	Ti-25Nb-3Fe-9Zr	Bal.	24.60 ± 1.8	3.92 ± 0.4	9.44 ± 1.0	935 ± 17.5
Ti30FZ	Ti-30Nb-3Fe-9Zr	Bal.	29.61 ± 4.2	3.41 ± 0.4	8.62 ± 1.4	902 ± 3.16
Ti35FZ	Ti-35Nb-3Fe-9Zr	Bal.	34.4 ± 3.3	2.42 ± 0.9	8.60 ± 0.6	794 ± 2.75

## Data Availability

The raw/processed data required to reproduce these findings cannot be shared at this time as the data also forms part of an ongoing research.
